# Seismological Processing of Six Degree-of-Freedom Ground-Motion Data

**DOI:** 10.3390/s20236904

**Published:** 2020-12-03

**Authors:** David Sollberger, Heiner Igel, Cedric Schmelzbach, Pascal Edme, Dirk-Jan van Manen, Felix Bernauer, Shihao Yuan, Joachim Wassermann, Ulrich Schreiber, Johan O. A. Robertsson

**Affiliations:** 1 Institute of Geophysics, ETH Zürich, 8092 Zürich, Switzerland; cedric.schmelzbach@erdw.ethz.ch (C.S.); pascal.edme@erdw.ethz.ch (P.E.); dirkjan.vanmanen@erdw.ethz.ch (D.-J.v.M.); johan.robertsson@erdw.ethz.ch (J.O.A.R.); 2 LMU, 80333 Munich, Germany; igel@geophysik.uni-muenchen.de (H.I.); fbernauer@geophysik.uni-muenchen.de (F.B.); syuan@geophysik.uni-muenchen.de (S.Y.); jowa@geophysik.uni-muenchen.de (J.W.); 3 Geodätisches Observatorium Wettzell, TUM, 93444 Bad Kötzting, Germany; ulrich.schreiber@tum.de

**Keywords:** seismology, rotation, gyroscope, ring laser, seismic tomography, seismic exploration

## Abstract

Recent progress in rotational sensor technology has made it possible to directly measure rotational ground-motion induced by seismic waves. When combined with conventional inertial seismometer recordings, the new sensors allow one to locally observe six degrees of freedom (6DOF) of ground-motion, composed of three orthogonal components of translational motion and three orthogonal components of rotational motion. The applications of such 6DOF measurements are manifold—ranging from wavefield characterization, separation, and reconstruction to the reduction of non-uniqueness in seismic inverse problems—and have the potential to revolutionize the way seismic data are acquired and processed. However, the seismological community has yet to embrace rotational ground-motion as a new observable. The aim of this paper is to give a high-level introduction into the field of 6DOF seismology using illustrative examples and to summarize recent progress made in this relatively young field. It is intended for readers with a general background in seismology. In order to illustrate the seismological value of rotational ground-motion data, we provide the first-ever 6DOF processing example of a teleseismic earthquake recorded on a multicomponent ring laser observatory and demonstrate how wave parameters (phase velocity, propagation direction, and ellipticity angle) and wave types of multiple phases can be automatically estimated using single-station 6DOF processing tools. Python codes to reproduce this processing example are provided in an accompanying Jupyter notebook.

## 1. Introduction

*“Instruments ought to be devised [...] to ends [...], such as the measurement of horizontal concussions, of vertical elevation, and of heaving or angular motion of the surface. It is no part of my present object to consider the probable movements of the soil in earthquakes. I limit myself to the description of a single instrument intended to measure lateral shocks, such as are experienced by objects placed upon a table which is abruptly shoved forwards.”*

**James David Forbes (1844)** [1].

After the occurrence of a series of pronounced earthquakes in Comrie in 1842, the British Association for the Advancement of Science awarded a grant of 20£ to form a committee to develop an instrument capable of registering the shocks of earthquakes [1,2]. This committee, led by Scottish physicist James David Forbes, constructed one of the first modern-day seismometers based on the principle of the inverted pendulum [3]. The instrument was capable of registering, and marking in pencil, horizontal translational displacements of the ground. Already then, Forbes and his co-investigator David Milne-Home acknowledged the necessity for additional instruments capable of measuring *‘heaving or angular motion of the surface’*.

Since then, the sensitivity of inertial seismometers measuring all of the three components of translational displacement (or temporal derivatives thereof, such as particle velocity or acceleration) has been constantly improving. Current day seismometers are capable of registering tiniest ground displacements of only a few nanometers, corresponding to about 1/100,000th of the width of a human hair. Signals recorded by such seismometers are routinely used by seismologists to image the Earth’s interior from the shallow subsurface down to the core. However, until recently, an instrument capable of recording angular ground motions, as envisaged by Forbes and Milne-Home, was still missing.

In fact, it took 150 years before Robert Nigbor could report on the first successful observations of such ‘rotational’ ground-motions using a solid-state rotational velocity sensor [4]. It was Nigbor who coined the term ’six-degree-of-freedom (6DOF) measurement’ for combined recordings of the three components of translational and the three components of rotational ground-motion. At this point, it might be important to state that a 6DOF measurement still does not provide a complete observation of seismically induced ground-motion. Only completely rigid objects (such as a seismometer or an airplane) are limited to six degrees of freedom. The Earth itself, however, is as an elastic continuum that can undergo deformations, such as dilational and shear strains. A 6DOF measurement is, therefore, still only a partial measurement. A complete observation of seismic ground-motion would require the additional recording of ground strains.

The sensitivity of the early solid-state rotational sensors used by Nigbor [4] and fiber-optical gyroscopes later used by Takeo [5] was limited to strong-motion signals recorded in the vicinity of artificial and earthquake sources.

The development of highly sensitive ring laser gyroscopes that were designed primarily with the aim to detect variations in the Earth’s absolute rotation rate led to further observations of earthquake-induced ground rotation [6,7]. However, the measurements were still not fully consistent in amplitude and phase with translational motions recorded with collocated seismometers. Thanks to further development of the ring laser technology, these inconsistencies could be eliminated [8,9,10,11].

Today, we are at a stage where rotational ground-motion observations are starting to become more widespread and more accurate. The first portable rotational sensors designed specifically for the needs of seismology have recently become available, e.g., blueSeis—3A by iXBlue; rotaphone [12,13]. Due to the increasing instrumentation coverage, the catalogue of rotational motion observations is continuously growing [14]. However, the seismological community yet has to embrace rotational motion as a new observable.

As we will try to lay out in this paper, the applications of rotational motion measurements are manifold—ranging from wavefield characterization, separation and reconstruction to the reduction of non-uniqueness in seismic inverse problems—and have the potential to revolutionize the way seismic data are acquired and processed. This paper aims to give a high-level introduction into the field of 6DOF seismology using illustrative examples and is intended for a reader with a general background in seismology.

This article is structured in the following way. We first revisit the fundamental theory underlying six degree-of-freedom data processing (Section 2). In Section 3, we then focus on the applications of combined rotation and translation measurements in seismology and provide two tutorials on how to process 6DOF data for applications, like wavefield characterization and wavefield reconstruction. Ultimately, an overview on the variety of rotation rate sensors and their working principles is given in Section 4.

For further information on the history of rotational seismology, we would like to refer the reader to the following reviews found in References [15,16,17,18,19,20,21].

## 2. Theoretical Foundations

**Figure 1:**
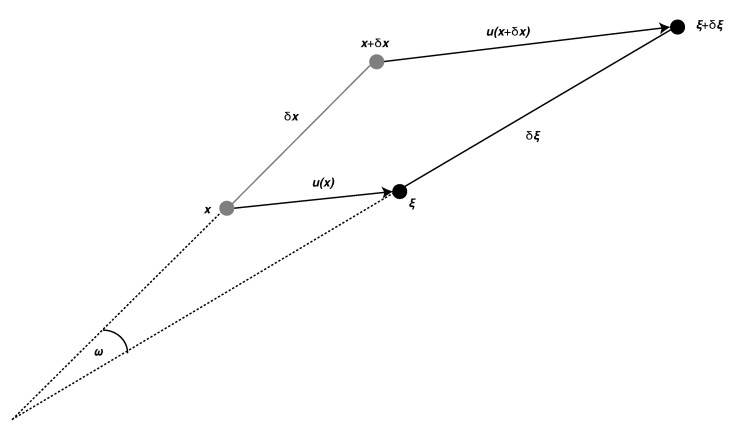
Displacement of a line segment δx with its end points x and x+δx in the undeformed state (light gray) being displaced by u(x) and u(x+δx) to ξ and ξ+δξ in the deformed state, respectively. The total rotation due to the displacement corresponds to the angle ω between δx and δξ modified after Reference [21].

In this section, we review the fundamental aspects of linear elasticity relevant to understand the rotational motions associated with seismic waves. In the following, we will work with a Cartesian coordinate system with coordinates *x*, *y*, and *z*. Consider two nearby points in 3-D space located at positions x and x+δx (Figure 1). Initially, the two points are separated by the distance δx. Now, suppose the point at x undergoes a displacement of u(x) moving it to ξ=x+u(x). In order to describe the deformation of the medium in the vicinity of x, we will consider the relative motion of the particle at x to a nearby point at x+δx, thus describing the deformation of the line segment δx to the deformed state δξ, (e.g., Reference [22]):

(1) δξ=δx+δu=x+δx+u(x+δx)−(x+u(x)).

We assume that |δx| is arbitrarily small so that u(x+δx) can be expanded as u(x)+(δx·∇)u using a first-order Taylor series. The relative displacement between the two points δu can thus be described by

(2) $$\delta u=\left(\begin{array}{c} \delta u_{x}\\ \delta u_{y}\\ \delta u_{z} \end{array}\right)=G\left(\begin{array}{c} \delta x\\ \delta y\\ \delta z \end{array}\right)=\left(\begin{array}{ccc} \partial_{x}u_{x} & \partial_{y}u_{x} & \partial_{z}u_{x}\\ \partial_{x}u_{y} & \partial_{y}u_{y} & \partial_{z}u_{y}\\ \partial_{x}u_{z} & \partial_{y}u_{z} & \partial_{z}u_{z} \end{array}\right)\left(\begin{array}{c} \delta x\\ \delta y\\ \delta z \end{array}\right),$$

where G is the displacement gradient tensor, which can be decomposed into symmetric and anti-symmetric parts as:

(3) $$G=\frac{1}{2}\left(G+G^{T}\right)+\frac{1}{2}\left(G-G^{T}\right).$$

If the displacement gradients are small ($|\partial_{j}u_{i}|\ll 1$), the symmetric part corresponds to the infinitesimal strain tensor

(4) $$\epsilon =\left(\begin{array}{ccc} \epsilon_{xx} & \epsilon_{xy} & \epsilon_{xz}\\ \epsilon_{yx} & \epsilon_{yy} & \epsilon_{yz}\\ \epsilon_{zx} & \epsilon_{zy} & \epsilon_{zz} \end{array}\right)=\left(\begin{array}{ccc} \partial_{x}u_{x} & \frac{1}{2}\left(\partial_{y}u_{x}+\partial_{x}u_{y}\right) & \frac{1}{2}\left(\partial_{z}u_{x}+\partial_{x}u_{z}\right)\\ \frac{1}{2}\left(\partial_{x}u_{y}+\partial_{y}u_{x}\right) & \partial_{y}u_{y} & \frac{1}{2}\left(\partial_{z}u_{y}+\partial_{y}u_{z}\right)\\ \frac{1}{2}\left(\partial_{x}u_{z}+\partial_{z}u_{x}\right) & \frac{1}{2}\left(\partial_{y}u_{z}+\partial_{z}u_{y}\right) & \partial_{z}u_{z} \end{array}\right),$$

and the anti-symmetric part corresponds to the infinitesimal rotation tensor

(5) $$\Omega =\left(\begin{array}{ccc} 0 & \frac{1}{2}\left(\partial_{y}u_{x}-\partial_{x}u_{y}\right) & \frac{1}{2}\left(\partial_{z}u_{x}-\partial_{x}u_{z}\right)\\ \frac{1}{2}\left(\partial_{x}u_{y}-\partial_{y}u_{x}\right) & 0 & \frac{1}{2}\left(\partial_{z}u_{y}-\partial_{y}u_{z}\right)\\ \frac{1}{2}\left(\partial_{x}u_{z}-\partial_{z}u_{x}\right) & \frac{1}{2}\left(\partial_{y}u_{z}-\partial_{z}u_{y}\right) & 0 \end{array}\right),$$

so that

(6) δu=ϵδx+Ωδx.

The infinitesimal rotation tensor can also be expressed in terms of the three-component rotation vector $\vec{\omega }$ so that

(7) $$\delta u=\epsilon \delta x+\vec{\omega }\times \delta x,$$

where the rotation vector is equal to half the curl of the displacement field:

(8) $$\vec{\omega }=\frac{1}{2}\nabla \times u=\frac{1}{2}\left(\begin{array}{c} \partial_{y}u_{z}-\partial_{z}u_{y}\\ \partial_{z}u_{x}-\partial_{x}u_{z}\\ \partial_{x}u_{y}-\partial_{y}u_{x} \end{array}\right).$$

It follows from Equation (8) that the rotation vector can be computed if the complete displacement field is known. On the other hand, missing information on the displacement field can be reconstructed if direct measurements of the rotation vector are available.

Under the assumption of infinitesimal displacement gradients, the second term in Equation (7) (ω×δx) is merely due to a static rotation and does not produce a deformation, e.g., Reference [22]. Therefore, it does not enter Hooke’s law. Within an elastic continuum, such as the Earth, there can be deformation, but no static rotation. Static rotations are purely associated with rotations of the whole continuum, for example due to the rotation of the Earth or the rotation of entire continents. For the time scales relevant in seismology, these effects are often negligible. In seismology, static rotations only occur in the near-field of large earthquakes. Recently, static rotations could be directly observed for the first time during an eruption of the Kilauea volcano using a dedicated rotational seismometer [23]. For weak motion (i.e., seismic waves), the displacement gradient of a deformation in an elastic continuum can be fully described by its symmetric part (i.e., the strain tensor). Yet, it is well known that the displacement gradient tensor of the seismic wavefield, unlike the deformations it causes, is not purely symmetric and that its rotation vector is not zero. In fact, if the displacement gradients of the seismic wavefield were purely symmetric, there would be no rotational ground-motion. To explain this apparent contradiction, we need to consider dynamic aspects in addition to the purely kinematic aspects discussed above.

For simplicity, we will consider a purely isotropic, homogeneous elastic medium. The strain tensor (describing the deformation of the medium, Equation (4)) can then be related to the stress tensor σ via the elastic constitutive equation (Hooke’s law)

(9) $$\sigma_{ij}=\left[\lambda \delta_{ij}\delta_{kl}+\mu \left(\delta_{ik}\delta_{jl}+\delta_{il}\delta_{jk}\right)\right]\varepsilon_{kl},$$

where λ and μ are Lamé’s constants, and $\delta_{ij}$ is Kronecker’s delta. Einstein’s summation convention is used for repeated subscripts, and it will be used in the following equations, as well. We now additionally consider the equation of motion

(10) $$\rho \partial_{t}^{2}u_{i}=f_{i}+\partial_{j}\sigma_{ij},$$

where $f_{i}$ denotes a body force, ρ is the density, and *t* is time. Substituting the constitutive equation (Equation (9)) and the definition of strain (Equation (4)) into the equation of motion (Equation (10)) yields the elastic wave equation, which can be written as:

(11) $$\rho \partial_{t}^{2}u=f+\left(\lambda +2\mu \right)\nabla \left(\nabla \cdot u\right)-\mu \nabla \times \left(\nabla \times u\right).$$

The wave equation describes the propagation of a displacement perturbation through an elastic, homogeneous medium. If we now take the curl of the elastic wave equation (Equation (11)) in a source-free region (i.e., $f_{i}=0$) and apply vector identities, we eventually find

(12) $$\nabla^{2}\left(\nabla \times u\right)-\frac{1}{\beta^{2}}\partial_{t}^{2}\left(\nabla \times u\right)=0,$$

where β is the shear wave velocity ($\beta =\sqrt{\mu /\rho }$). Equation (12) is simply the wave equation applied to the curl of the displacement field with a velocity of β. In an isotropic homogeneous elastic medium, Equation (12) describes the propagation of S-waves. Equation (12) demonstrates that, in an isotropic medium, the curl of an elastic wavefield, and thus the anti-symmetric part of the displacement gradient tensor (Equation (7)) measured by a rotational sensor, is indeed not zero but equal to the shear wave constituent of the wavefield.

This becomes more clear when we consider a simple solution to the elastic wave equation in the form of the plane-harmonic solution for waves so that the particle velocity (temporal derivative of displacement) at position $r={\left[x,y,z\right]}^{T}$ and at time *t* is described by $\dot{u}\left(r,t\right):R^{4}\to R^{3}$ as:

(13) $$\dot{u}\left(r,t\right)=Ah\cos\left(\omega t-k\cdot r\right),$$

where *A* is the particle velocity peak amplitude, h is the polarization vector describing the direction of the particle displacement, and ω is the angular frequency. The vector quantity k=kl is the wave vector in which magnitude *k* is the wavenumber, which is related to the propagation velocity *c* of the wave as k=ω/c. The vector quantity $l={\left[l_{x},l_{y},l_{z}\right]}^{T}$ determines the propagation direction of the wave by the direction cosines $l_{i}$.

If we now estimate the rotational motions of this wave by applying Equations (8)–(13), we find the rotation rate vector:

(14) $$\vec{\dot{\omega }}\left(r,t\right)=\frac{1}{2}\left(\nabla \times \dot{u}\right)=-\frac{A}{2}k\times h\sin\left(\omega t-k\cdot r\right)=-\frac{A}{2}\frac{\omega }{c}\left(\begin{array}{c} l_{y}h_{z}-l_{z}h_{y}\\ l_{z}h_{x}-l_{x}h_{z}\\ l_{x}h_{y}-l_{y}h_{x} \end{array}\right)\sin\left(\omega t-k\cdot r\right).$$

From Equation (14), it follows that P-waves inside an isotropic medium do not generate rotational motions since, for P-waves, the polarization vector is parallel to the propagation direction so that h=l and thus $\vec{\dot{\omega }}=0$. S-waves, on the other hand, exhibit ground displacements perpendicular to the propagation direction, thereby causing a simple shear deformation and thus generate rotational motions. Equation (14) states that rotational motions are only generated by waves that exhibit particle displacements nonparallel to the propagation direction. In anisotropic media, where both the polarization vector h and the velocity *c* depend on the wave propagation direction l, P-waves can thus also generate rotational motions [24].

Another key observation that follows directly from Equation (14) is that the amplitude of the rotational motions induced by a plane wave inversely scale with the wave’s phase velocity *c*. If we now take the temporal derivative of Equation (13), thereby converting the particle velocities to particle acceleration, we obtain:

(15) $$\ddot{u}\left(r,t\right)=-A\omega h\sin\left(\omega t-k\cdot r\right).$$

This result is of specific interest since it illustrates one of the key benefits of combined rotation and translation measurements. If both quantities are measured at a discrete point in space r, the ratio between the magnitude of the two measurements yields

(16) $$\frac{|\ddot{u}\left(r,t\right)|}{|\vec{\dot{\omega }}\left(r,t\right)|}=2c{\left|l\times h\right|}^{-1},$$

where the magnitude of the cross product on the right hand side is 1, if l⊥h (i.e., for an S-wave in an isotropic medium). In this case, the ratio between the two measurements at a single point provides direct access to the shear wave speed, a quantity that can conventionally only be obtained from the analysis of array data. On the other hand, the magnitude of the cross product on the right hand side of Equation (16) becomes 0, if l ‖ h (i.e., for a P-wave in an isotropic medium). In the case of an anisotropic medium, the magnitude of the cross product can take on any value between 0 and 1, depending on the angle between the propagation and polarization directions.

### Rotational Ground Motion at the Free Surface

Most seismic measurements are taken directly at or immediately below the Earth’s surface. This has direct implications on the quantities that are measured by a rotational seismometer due to the special boundary conditions that apply. At a free surface, all stresses acting in the direction normal to the surface vanish (zero-traction boundary condition). Revisiting the stress-strain relationship for an elastic, isotropic medium (Equation (9)), we find the following conditions for the stress rate components ${\dot{\sigma }}_{xz}$, ${\dot{\sigma }}_{yz}$, and ${\dot{\sigma }}_{zz}$ at the Earth’s surface:

(17) $$\begin{array}{c} {\dot{\sigma }}_{xz}=\mu (\partial_{x}{\dot{u}}_{z}+\partial_{z}{\dot{u}}_{x})=0,\\ {\dot{\sigma }}_{yz}=\mu (\partial_{z}{\dot{u}}_{y}+\partial_{y}{\dot{u}}_{z})=0,\\ {\dot{\sigma }}_{zz}=\lambda \left(\partial_{x}{\dot{u}}_{x}+\partial_{y}{\dot{u}}_{y}+\partial_{z}{\dot{u}}_{z}\right)+\mu \left(\partial_{z}{\dot{u}}_{z}+\partial_{z}{\dot{u}}_{z}\right)=0. \end{array}$$

At the free surface, the vertical derivatives can thus be expressed in terms of horizontal derivatives as:

(18) $$\begin{array}{c} \partial_{z}{\dot{u}}_{x}=-\partial_{x}{\dot{u}}_{z},\\ \partial_{z}{\dot{u}}_{y}=-\partial_{y}{\dot{u}}_{z},\\ \partial_{z}{\dot{u}}_{z}=-\frac{\lambda }{\lambda +2\mu }\left(\partial_{x}{\dot{u}}_{x}+\partial_{y}{\dot{u}}_{y}\right). \end{array}$$

As a result, the rotation vector (Equation (8)) at the Earth’s surface can be expressed using purely horizontal derivatives in the following form:

(19) $$\vec{\dot{\omega }}=\frac{1}{2}\nabla \times \dot{u}=\left(\begin{array}{c} \partial_{y}{\dot{u}}_{z}\\ -\partial_{x}{\dot{u}}_{z}\\ \frac{1}{2}\left(\partial_{x}{\dot{u}}_{y}-\partial_{y}{\dot{u}}_{x}\right) \end{array}\right).$$

This means that a rotation sensor placed at the Earth’s surface provides a way to directly measure the horizontal derivatives of the vertical wavefield component. This result can be used to relax spatial sampling requirements in seismic exploration by the use of generalized sampling concepts (see Section 3.2). Additionally, Equation (19) implies that two-dimensional surface-deployed seismometer arrays are sufficient to geodetically derive the full rotation vector at the Earth’s surface (no buried sensors are required to measure the vertical derivatives) above a homogeneous and isotropic medium (see Section 4.2).

Another effect of the Earth’s surface is that there will be rotational motions associated with the arrival of a P-wave, even in an isotropic medium. This is due to mode-conversions that take place at the boundary. Incoming P-waves at the Earth’s surface reflect into both a down-going P-wave and an additional down-going S-wave. The rotational motion generated by this down-going S-wave will be picked up by a rotational sensor. The relative amplitude of the reflected wave (and thus the amount of rotational motion generated) depends on the incident angle of the incoming wave and the local P- and S-wave velocities at the recording station. Low velocities and incident angles close to the horizontal will yield larger amplitudes in rotational motions. A single 6DOF measurement can therefore theoretically be used to directly constrain the local P- and S-wave velocities [25].

## 3. Processing of 6DOF Ground-Motion Measurements

Early research on potential applications of rotational motion measurements was primarily driven by the earthquake seismology and earthquake engineering communities. This is mainly due to the rapid development of the ring laser technology, which enabled the direct observation of rotational motions in the frequency-band relevant for earthquake seismology. Applications to seismic exploration problems were hampered by the lack of a portable, affordable sensor with the required sensitivity. However, some applications on the exploration scale have been demonstrated using array-derived estimates of rotational motion. In the following, we will give an overview of the current state of research in analyzing 6DOF recordings both on the global and the exploration scale with a particular focus on progress made in the past few years.

### 3.1. Array-Like Capabilites of Single-Station 6DOF Measurements

Since the 1960s, seismology has profited immensely from the application of array processing methods. Arrays of seismometers with a uniform instrument response (or appropriately corrected response) enable the direct observation of the propagation delays of seismic phases as they pass through the array, thereby providing a way to extract the vector velocity (wave speed and propagation direction) of an incident wavefront by the application of array processing techniques, such as beamforming, slant-stacking, or frequency-wavenumber analysis [26]. One of the downsides of seismological arrays is that they are expensive to deploy, and special care needs to be taken to ensure that the network is homogeneous and that site effects are avoided.

Since single-station 6DOF recordings provide direct access to the phase velocity and propagation direction under the plane wave assumption (Equation (16)), they theoretically provide similar capabilities in extracting the vector velocity as seismological arrays, which is why 6DOF recording stations are sometimes referred to as *‘point seismic arrays’* [27]. The rich amount of information that can be extracted from 6DOF recordings makes such ‘point arrays’ attractive for the deployment in areas where extended receiver arrays cannot be used, for example, on the ocean bottom [28], in mountainous areas, or in extraterrestrial seismology [29].

#### 3.1.1. Single-Station 6DOF Wave Parameter Estimation

The potential to extract the vector velocity of seismic phases was recognized early on as one of the key benefits of rotation measurements for seismology [6,7,30]. Due to significant advances in the accuracy of ring laser measurements, this capability could be demonstrated on 6DOF data for Love waves of teleseismic and regional earthquakes [9,31,32,33]. The noise floor of modern-day ring lasers (down to $10^{-12}$ rad/s over a wide frequency-range) is even low enough to observe ocean-generated microseismic noise. This allowed Hadziioannou et al. [34] to extract the phase velocity and back-azimuth of the strongest sources in the secondary microseismic noise band. The extracted wave parameters were consistent with estimates obtained from conventional array processing.

For sources that generate broad-band energy, a single 6DOF observation enables the estimation of frequency-dependent phase velocities (dispersion curves), which can be used to extract depth-dependent velocity profiles [35,36,37,38,39,40,41].

Marano and Fäh [42] proposed a maximum likelihood method for the estimation of wave parameters using 6DOF sensors and showed that rotational motion data help to resolve some of the ambiguity in the single-station seismic direction finding problem.

Sollberger et al. [25] recognized that tools from conventional three-component polarization analysis can be adapted to 6DOF data (so-called six-component polarization analysis), which enables the single-station estimation of wave parameters. They showed that some of the fundamental limitations of single-station three-component polarization analysis can potentially be overcome with six-component polarization analysis including the estimation of wave parameters for multiple events overlapping in time, the 180 degrees ambiguity in estimation of the back-azimuth, the recovery of the true incidence angle of body waves at the free surface, and the unambiguous identification of the wave type.

Rotational motions are direct measurements of the anti-symmetric part of the gradient tensor of the displacement field (Section 2). Hence, the extraction of wavefield properties by the combined analysis of rotational and translational motion is closely related to the so-called wave gradiometry technique [43,44,45,46]. Wave gradiometry aims to extract wavefield properties, such as phase velocity, back-azimuth, geometrical spreading, and radiation pattern, by the combined analysis of the wavefield and its spatial derivatives (i.e., the wavefield gradient). The spatial derivatives are thereby estimated from dense receiver arrays using a finite-difference approximation.

Similarly, the second-order spatial derivatives of the wavefield can be estimated using a finite-difference approximation, so that one can directly invert the hyperbolic wave equation for local material properties [47]. De Ridder and Biondi [48] demonstrated that such methods, which are closely related to the wave gradiometry technique, can be successfully applied to short recordings of ambient seismic noise to image the local velocity structure without the need to compute cross-correlations of long noise records. De Ridder and Curtis [49] extended the method to also yield anisotropic wavefield parameters.

#### 3.1.2. Single-Station 6DOF Wave Mode Filtering

Conventionally, the decomposition of a recorded seismic wavefield into different wave modes (P-, S-, or surface waves) can only be achieved when processing array data. Different phases are thereby identified based on their different moveout velocities along extended receiver arrays and then separated using techniques, such as f-k- or slant-stack filtering, e.g., Reference [50]. By additionally taking into account polarization information provided by three-component translational recordings, a better separation between different phases can be achieved, e.g., Reference [51,52]. Nevertheless, at a single recording station, pure translational motions are not indicative of wave type (e.g., P- and S-waves both exhibit a rectilinear polarization on the three translational components). In contrast, rotational motion recordings are direct measurements of the wavefield’s curl and are thus exclusive to S-waves and surface waves in isotropic media (see Section 2). Rotational motion measurements can thus highly facilitate the identification and isolation of different wave modes in seismic data. This is of particular importance in seismic exploration where the recorded wavefields are generally complex due to the heterogeneous near-surface zone and short source-receiver offsets, causing different wave-modes to severely interfere and overlap in time, thereby exacerbating a separate interpretation.

Several studies have used the wave mode filtering properties of rotational motions to improve the S-wave interpretation of seismic recordings. Robertsson and Muyzert [53] proposed to employ small-aperture 3D arrays of three-component geophones in a tetrahedral shape (three surface geophones in a triangle and a buried geophone in the center) within which the curl (rotation) and divergence operators can be estimated in order to separate S-, and P-waves. By exploiting the free-surface boundary condition, Robertsson and Curtis [54] derived expressions to compute the curl and divergence operators using purely surface-deployed geophone arrays (no buried geophone required). The authors suggested to additionally apply up-/down- separation filters based on the elastodynamic representation theorem to suppress mode-converted waves at the free surface. Compact, truncated expressions for these spatially infinite filters were recently proposed by Van Renterghem et al. [55] by incorporating spatial wavefield derivatives (and thus rotational motion measurements). These filters allow one to separate the wavefield into its up-/down-going and P-/S-constituents for a limited range of incidence angles.

Woelz et al. [56] conducted a high-resolution 3D seismic survey over a site of archaeological interest and successfully isolated the SH-constituent of the recorded wavefield by estimating the vertical component of the rotation vector using a finite-difference approximation between closely spaced geophones. Sollberger et al. [29] used estimates of rotational motion to identify S-waves in the complex seismograms recorded on the lunar surface during the Apollo 17 mission and could extract the first comprehensive elastic parameter model of the shallow lunar subsurface. Rotational motions were thereby array-derived from a small-aperture triangular array of purely vertical-component geophones by exploiting the free surface boundary condition (Equation (19)).

Since theoretically, the vertical component of rotation is only sensitive to Love and SH-wave motion in isotropic media, Tanimoto et al. [57] could successfully estimate the Rayleigh-to-Love wave energy ratio in the secondary microseism by comparing this component with the vertical translational ground acceleration recorded on a conventional seismometer, which is insensitive to Love waves. In a similar fashion, Chow et al. [58] used the vertical component of rotation to characterize the amplitude decay of Love waves. Yuan et al. [59] exploits the fact that the two horizontal components of rotation are purely sensitive to SV- and Rayleigh-wave motion, thereby providing a stable way to estimate the back azimuth of seismic arrivals with a single station that does not suffer from the interference of different modes of vibration. In this way, a single 6DOF recording station can be used to effectively track the evolution of earthquake ruptures in real time [60].

Sollberger et al. [25] demonstrated that, at the free surface, the 6DOF ground motion pattern of plane waves is unique for each wave type (P-, SV-, SH/Love-, and Rayleigh-waves) and that the wave types of two interfering arrivals can be estimated simultaneously using a single 6DOF station. This allows one to automatically separate the recorded data into contributions from different wave types (see the example given in Section 3.1.3).

The wave mode filtering aspect of rotational motion data is also one of the key benefits of such measurements for land seismic exploration, besides the wavefield reconstruction capabilities discussed in Section 3.2. Surface waves (in the exploration community often referred to as ground-roll) cause a major problem in land-seismic exploration since they often cover up the underlying reflection signal that carries the desired information on subsurface resources. Conventional filtering techniques that aim to suppress ground roll in the recordings require the spatial sampling to be smaller than half the horizontal wavelength of the surface waves (Shannon-Nyquist sampling). Since surface waves typically exhibit the shortest horizontal wavelengths in the entire recording, the spatial sampling in land seismic exploration is often dictated by this undesired part of the signal. As a result, land seismic exploration campaigns involve an enormous amount of sensors and channels, causing them to be extremely costly operations.

With 6DOF recordings, surface waves can be identified and suppressed using just a single station without any requirements on spatial sampling [61,62]. Since the amplitude of rotational motions inversely scale with phase velocity (e.g., Equation (14)), the low-velocity surface waves appear much stronger in rotational data than the high-velocity body-wave reflection signal. By adaptively subtracting the rotational signal from the translational vertical component data (e.g., vertical component geophone), surface waves can be effectively suppressed [63]. If the inline horizontal-component of ground rotation is measured, even side-scattered surface waves can be suppressed, which pose a major problem to land seismic imaging, especially in karstified and desert areas [64]. Sollberger et al. [65] developed a fully automatic ground-roll identification and suppression scheme, that also successfully removes side-scattered surface waves, based on the characteristic 6DOF ground motion pattern exhibited by surface waves. The method retains most of the underlying reflection signal.

#### 3.1.3. Example: Tutorial on 6DOF Processing Using the 2018 Gulf of Alaska Earthquake

**Figure 2:**
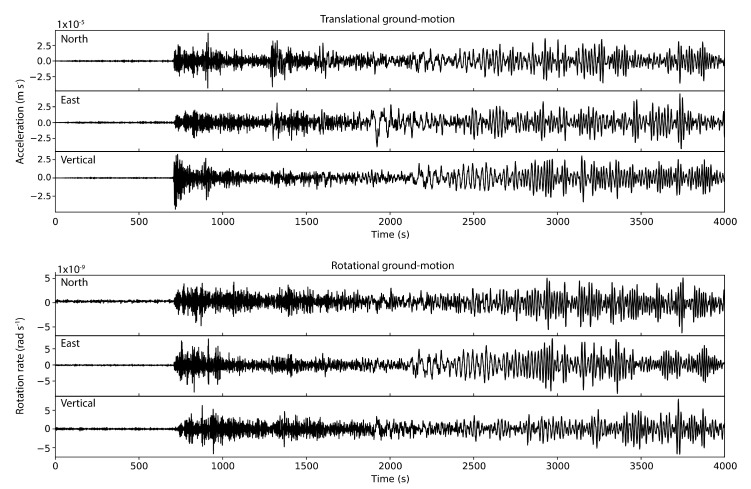
Six-degree-of-freedom (6DOF) recording of the M7.9 23 January 2018, gulf of Alaska earthquake (occurence time: 2018-01-23 09:31:40 UTC) at the geophysical observatory in Fürstenfeldbruck, Germany. Rotational data were recorded by the large multi-component ring laser ROMY and translational data were recorded by a collocated seismometer.

On 23 January 2018, a magnitude 7.9 earthquake occurred in the gulf of Alaska. Seismic waves of the event were recorded on the newly-opened multi-component ring laser observatory ROMY in Fürstenfeldbruck, Germany [66,67], resulting in the first direct observations of all six degrees of freedom of ground motion of a teleseism. The recorded data is shown in Figure 2. The data is low-pass filtered so that it only contains frequencies up to 1 Hz. Here, we will illustrate the array-like processing that can be applied to this single-station data in order to extract wave parameters and to estimate the wave type of different arrivals. This section should serve as a short introductory tutorial into 6DOF data processing. Data and Python codes to reproduce the processing examples that are discussed in this section are provided alongside this paper and can be accessed at https://github.com/solldavid/6DOF_processing_tutorial. Please refer to the Jupyter Notebook provided in this repository for instructions on how to use the codes.

**Figure 3:**
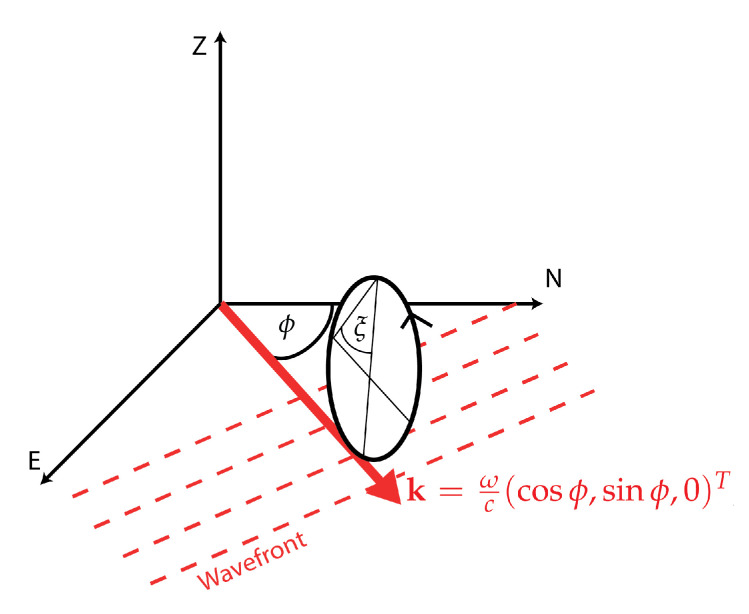
Schematic drawing of the plane harmonic model used to describe Rayleigh waves for single-station 6DOF analysis.

Firstly, we are interested in extracting wave parameters, such as the back-azimuth and the phase velocity of seismic arrivals, from the six-component time series. For illustration purposes, we will only focus on a single wave type. Here, we choose to analyze Rayleigh waves, yet the same type of processing can be applied to extract wave parameters for all wave types. Let us start by revisiting the mathematical model describing the 6DOF ground-motion associated with the arrival of a plane Rayleigh wave at a recording station. This is important to understand the range of parameters that can be constrained with a 6DOF recording. Again, we will use the harmonic plane-wave approximation given in Equation (13). The wave vector of a Rayleigh wave is purely restricted to the horizontal plane since the wave propagates along the surface and is given by $k=k{\left(\cos\phi ,\sin\phi ,0\right)}^{T}$, with ϕ being the propagation azimuth. The magnitude of the wave vector *k* is described by $k=\frac{\omega }{c}$, with ω the angular frequency, and *c* the Rayleigh wave phase velocity. Rayleigh waves exhibit an elliptical particle motion in the vertical plane, which can be described by the polarization vector $h={\left(\sin\xi \cos\phi ,\sin\xi \sin\phi ,\cos\xi \right)}^{T}$, where ξ is called the ellipticity angle of the Rayleigh wave, determining the eccentricity and the sense of rotation (prograde or retrograde) of the particle motion. An illustration of this simple model is depicted in Figure 3.

Inserting these expressions into the plane-harmonic solution in Equation (13) thus yields the following model for the North-, East-, and vertical-components of particle velocity ${\dot{u}}_{n}$, ${\dot{u}}_{e}$, and ${\dot{u}}_{z}$, respectively, associated with the arrival of a Rayleigh wave:

(20) $$\begin{array}{c} {\dot{u}}_{n}\left(r,t\right)=A\cos\phi \sin\xi \cos\left(\omega t-k\cdot r\right),\\ {\dot{u}}_{e}\left(r,t\right)=A\sin\phi \sin\xi \cos\left(\omega t-k\cdot r\right),\\ {\dot{u}}_{z}\left(r,t\right)=A\cos\xi \cos\left(\omega t-k\cdot r+\pi /2\right). \end{array}$$

Note that the vertical component is phase-shifted by π/2 with respect to the horizontal components, causing the characteristic elliptical particle trajectory.

The rotational motions associated with the arrival of this wave can now be found by computing the free-surface rotation vector (Equation (19)), yielding the following rotation rates:

(21) $$\begin{array}{c} {\dot{\omega }}_{n}=A\frac{\omega }{c}\sin\phi \cos\xi \cos\left(\omega t-k\cdot r\right)\\ {\dot{\omega }}_{e}=-A\frac{\omega }{c}\cos\phi \cos\xi \cos\left(\omega t-k\cdot r\right)\\ {\dot{\omega }}_{z}=0. \end{array}$$

This result shows that rotational motions induced by a Rayleigh wave are limited to rotations around the two horizontal axes. Linearly polarized Rayleigh waves (i.e., ξ=±π/2) do not produce any rotational motion.

For the joint analysis of translational and rotational data, it makes sense to convert the particle velocities in Equation (20) to acceleration. The reasoning behind this is that the amplitudes of rotational motions scale with frequency (see the factor ω in Equation (21)), whereas this is not the case for the translational motions. If we take the temporal derivative of the particle velocities in Equation (20), we obtain

(22) $$\begin{array}{c} {\ddot{u}}_{n}\left(r,t\right)=-A\omega \sin\xi \cos\phi \sin\left(\omega t-k\cdot r\right),\\ {\ddot{u}}_{e}\left(r,t\right)=-A\omega \sin\xi \sin\phi \sin\left(\omega t-k\cdot r\right),\\ {\ddot{u}}_{z}\left(r,t\right)=-A\omega \cos\xi \cos\left(\omega t-k\cdot r\right), \end{array}$$

where the amplitudes of the acceleration now also scale with frequency. As a result, the relative amplitudes of the wave on the all six components (translational acceleration and rotation rate) can be described by a single six-component vector v, where the first three components describe the amplitude of the wave in the translational motions and the rest describe the amplitude of the wave in the rotational motions:

(23) $$v=A\omega \left(\begin{array}{c} -j\sin\xi \cos\phi \\ -j\sin\xi \sin\phi \\ -\cos\xi \\ c^{-1}\cos\xi \sin\phi \\ -c^{-1}\cos\xi \cos\phi \\ 0 \end{array}\right)$$

Here, *j* denotes the imaginary unit, indicating that the two horizontal translational components are phase-shifted by π/2 with respect to the other components. Note that the amplitude and frequency of the wave only affect the magnitude of this vector quantity, yet the direction of the vector is independent of amplitude and frequency and can be fully described by the propagation azimuth ϕ, the ellipticity angle ξ, and the phase velocity *c* of the Rayleigh wave. Therefore, it should be possible to extract these wave parameters by simply analyzing the relative amplitudes of a wave recorded on a single 6DOF station. Sollberger et al. [25] refer to the direction of the vector v as the six-component polarization direction of a Rayleigh wave. The aim is now to try and parameterize this model such that it optimally fits the polarization direction present in the actual, recorded data. In the following, this process is referred to as 6DOF polarization analysis.

Let us now return to the 6DOF data recorded after the gulf of Alaska earthquake (Figure 2). We could try to simply fit the direction of the polarization vector in Equation (23) to the direction described by the six samples at each sampling location of the time series in order to estimate Rayleigh wave parameters. Clearly, this would not lead to any stable results, mainly for two reasons.

Firstly, the recorded time series is highly oscillatory and includes many zero-crossings where the amplitudes of the recorded wave drop below the noise level, resulting in very poor estimates of the wave parameters.

Secondly, the polarization direction model described by Equation (23) is only valid for a single Rayleigh wave. Yet, we know that the recorded data also includes P-, S-, and Love waves that could potentially interfere in time. The extracted wave parameters would thus only have very limited meaning.

Since we know that seismic waves are transient events of limited duration, it is thus advisable to perform the analysis in a limited time window within which the signal-to-noise ratio can be improved and where optimally only a single Rayleigh wave is present. If the time window is chosen appropriately, and only a single Rayleigh wave is present in the analysis window, the complete 6DOF ground-motion in the window will be along a single direction which can be described by the polarization vector in Equation (23). However, it turns out that performing the analysis in a time window is often still not enough to achieve a stable result. The reason is that the direction of the polarization vector in Equation (23) is only seemingly independent of frequency. It is well-known that the phase velocity *c* of Rayleigh waves is strongly frequency-dependent itself due to the layering in the Earth’s crust so that c=c(ω). Better results can thus be achieved by performing the analysis on a time-frequency decomposed representation of the time-series [68]. Another benefit of performing a time-frequency decomposition is that an additional separation of differrent wave modes can be achieved, making the data better suited for fitting a model of just a single wave type.

**Figure 4:**
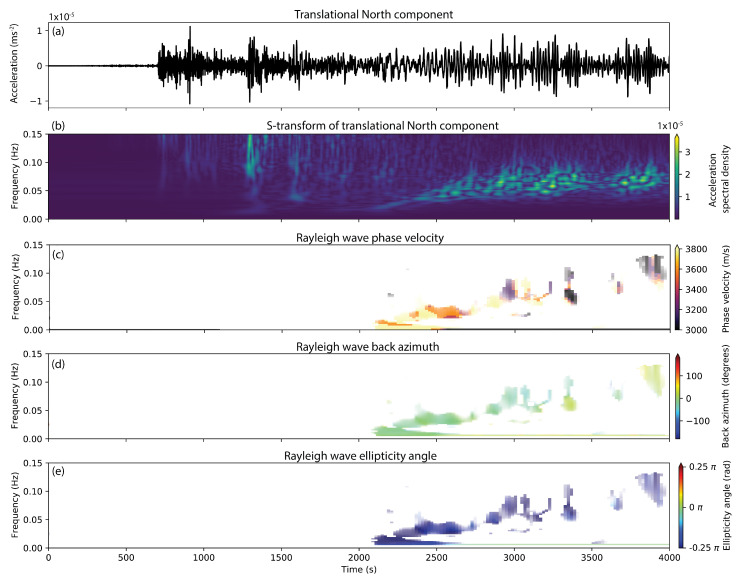
Single-station wave parameter estimation using 6DOF polarization analysis on the example of the 2018 gulf of alaska earthquake. The North component of translational motions is displayed in (**a**) with the corresponding S-transform in (**b**). Shown below is the estimation of frequency- and time-dependent Rayleigh wave parameters: phase velocity (**c**), back azimuth (**d**), and ellipticity angle (**e**). The results are only displayed at time-frequency pairs where the automatic detection routine identified a Rayleigh wave (Figure 5). See text for details.

Figure 4a shows the North component (which is roughly in the radial direction) seismogram of translational motion of the Alaska earthquake. A time-frequency decomposed representation of the same seismogram obtained by computing the S-transform [69] is shown in Figure 4b. The early arriving phases in the frequency band above 0.05 Hz correspond to body waves (P- and S-phases). The later-arriving surface waves (on the North component mainly Rayleigh waves) generally show a lower frequency content.

Now, we try to fit our wave model to the recorded data in order to find the best-fitting wave parameters, which are, for a Rayleigh wave: the phase velocity, the azimuth and the ellipticity. There are multiple ways to achieve this, among the methods that have been proposed are the maximum likelihood estimator [42], orthogonal distance regression [36], and the MUSIC algorithm [25]. It was shown that, due to the extension of the data dimensionality introduced by the additional rotational data, parameters of two interfering arrivals can be simultaneously estimated [25], which is not possible with conventional three-component polarization analysis. Here, we choose a simple approach of estimating the wave parameters: We try to minimize the angle between the vector describing our theoretical model (Equation (23)) and the vector describing the dominant polarization direction in the data. The best-fitting wave parameters are found, when the two vectors are parallel, which means that the angle between the two is zero.

A common way to extract the polarization direction from multicomponent data at each time-frequency pair (ω,t), is to perform an eigenanalysis of the so-called spectral matrix S(ω,t) [70]. The 6 × 6 spectral matrix is formed within a time-frequency window by averaging the outer product of the data vector with itself:

(24) $$S\left(\omega ,t\right)=g\left(\Delta \omega ,\Delta t\right)*u\left(\omega ,t\right)u^{H}\left(\omega ,t\right).$$

Here, $u\left(\omega ,t\right)={\left({\ddot{u}}_{n},{\ddot{u}}_{e},{\ddot{u}}_{z},{\dot{\omega }}_{n},{\dot{\omega }}_{e},{\dot{\omega }}_{z}\right)}^{T}\left(\omega ,t\right)$ is a vector composed of the time-frequency decomposed 6DOF data, * denotes convolution, and ${\left(.\right)}^{H}$ is the Hermitian conjugate transpose. The function g(Δω,Δt) is a windowing function of spectral width Δω and temporal width Δt, for example, a Gaussian window.

An eigendecomposition of the spectral matrix will then reveal the dominant polarization direction of the data as the eigenvector $e_{1}$ associated with the largest eigenvalue.

At each time-frequency pair, we now try to find the parameter vector m(ω,t)=(ϕ,ξ,c)(ω,t) that minimizes the angle φ(m) between the modeled polarization vector v(m,ω,t) in Equation (23) and the dominant polarization vector in the data $e_{1}\left(\omega ,t\right)$, where the angle is given by the dot product:

(25) $$\cos\varphi =\frac{v\left(m,\omega ,t\right)\cdot e_{1}\left(\omega ,t\right)}{\left|v(m,\omega ,t)\right|\left|e_{1}\left(\omega ,t\right)\right|}.$$

The set of best-fitting wave parameters $\hat{m}\left(\omega ,t\right)$ can now be found by numerically maximimizing the following likelihood function by a grid search:

(26) $$\hat{m}\left(\omega ,t\right)=\underset{m(\omega ,t)}{\arg\max}\;\left(e^{-\varphi^{2}\left(m\right)}\right).$$

The exponential in Equation (26) will take on a maximum value of 1 when the modeled polarization vector is parallel to the data polarization vector (i.e., when φ(m)=0, and a perfect data fit is found) and will decay exponentially away from the maximum.

The results of applying this wave parameter estimation scheme to the 6DOF signal recorded after the gulf of Alaska earthquake are given in Figure 4c–e. Shown are the color-coded, estimated Rayleigh wave parameters $\hat{m}\left(\omega ,t\right)$ that best fit the observed data. Values are only displayed for time-frequency pairs where the maximum of the likelihood function (exponential in Equation (26)) reached values larger than 0.7. For the computation of the spectral matrix, we used a Gaussian time window that extends over two dominant periods in time (frequency-dependent) and over 0.01 Hz in frequency.

Several interesting observations can be made when analyzing this result. The estimated phase velocities show the typical dispersion characteristic that is expected for surface wave arrivals. Events with high phase velocity arrive first at lower frequencies (sensing deeper parts of the Earth). At later times, the phase velocity then seems to decrease with a trend towards higher frequencies. This highlights the potential of single-station 6DOF measurements to extract dispersion curves. Stable results seem to be obtained for both the back-azimuth and the ellipticity angle. Note that the back-azimuth can be resolved on the full 360 degrees interval and does not show the typical 180 degree ambiguity inherent in conventional three-component polarization analysis. The extracted back-azimuth at low frequencies shows an average value of −15 degrees, which nicely corresponds to the theoretical back azimuth for the gulf of Alaska earthquake (theoretical azimuth: −11 degrees). At higher frequencies the back-azimuth seems to slightly deviate from the theoretical back azimuth and reaches values between −30 and +20 degrees. This deviation could potentially be caused by 3D structure in the Earth’s crust. The estimated ellipticy angles show constant values close to −0.25π, indicating the typical retrograde elliptical motion associated with the arrival of a Rayleigh wave.

Additionally, the wave-mode filtering capabilities of 6DOF measurements also become apparent when looking at the results in Figure 4. As mentioned above, wave parameters are only plotted where the best-fitting wave parameter model resulted in a likelihood value larger than 0.7 in the estimator described in Equation (26). The large blank areas in Figure 4c–e indicate that for the early body-phase arrivals, the Rayleigh wave model only poorly fits the data. The reason for this is that the 6DOF ground-motion pattern is unique for each wave type [25], meaning that, if another wave type is dominant in the analysis time-frequency window, no parametrization of the Rayleigh wave 6DOF polarization vector v exists that causes it to lie parallel to the dominant polarization direction in the data $e_{1}$.

**Figure 5:**
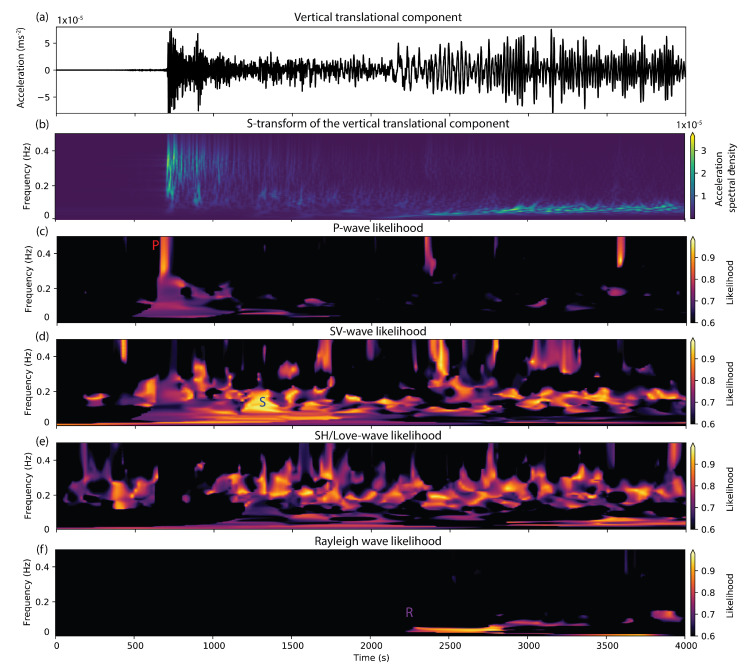
Automated wave type classification using 6DOF polarization analysis. High likelihood values indicate where the 6DOF polarization template for a specific wave type matches the polarization present in the data. Since the 6DOF polarization templates are unique for each wave type, this enables the automatic classification of different phases using just a single 6DOF recording. The vertical component translational seismogram and its S-transform are shown in (**a**,**b**). Likelihood values obtained from 6DOF analysis are shown in (**c**) for P-waves, (**d**) for SV-waves, (**e**) for SH/Love-waves, and (**f**) for Rayleigh waves.

Sollberger et al. [25] provide 6DOF polarization vectors for all wave types that can be fitted to match the direction of $e_{1}$ using the same estimation scheme that is described above. The results of performing such an analysis are shown in Figure 5. The first two panels show the vertical component seismogram of translational motions of the gulf of Alaska earthquake (Figure 5a) and its S-transform (Figure 5b) up to frequencies of 0.5 Hz. The following panels show the color-coded likelihood values (value of the exponential in the estimator described in Equation (26)) for the best-fitting polarization models of the four wave types P, SV, SH/Love, and Rayleigh. High values indicate that the polarization model fits the data at a specific time-frequency pair. Since the 6DOF polarization models are unique for each wave type, the results can be used to automatically classify the different arrivals in terms of their wave type. Several arrivals seem to be classified correctly by the algorithm, such as the direct P wave (marked in red in Figure 5c), the direct S-wave (marked in blue in Figure 5d, and the Rayleigh waves (Figure 5f, as already discussed above). Note that a significant amount of S-wave energy seems to be detected by the algorithm in between the direct P- and the direct S-phase. This could be explained by P-to-S-scattering in the Earth’s crust.

**Figure 6:**
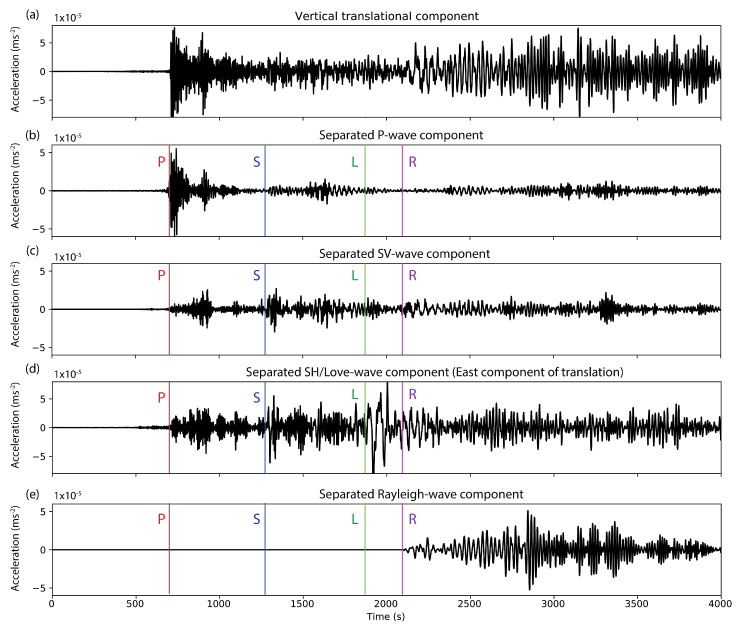
Single-station 6DOF wave-mode filtering applied to the gulf of Alaska earthquake. The automatic wave-mode classification of different arrivals shown in Figure 5 can be used to filter the data to yield wave-type specific seismograms. The original vertical component of translational motion is displayed in (**a**). The panels (**b**–**e**) show the output of the proposed wave-mode separation filters for P-waves (**b**), SV-waves (**c**), SH-/Love waves (**d**), and Rayleigh waves (**e**). Colored lines mark arrival times of some key phases obtained from ray tracing.

The likelihood values displayed in Figure 5 can also be used to filter data by simply multiplying them with the S-transformed data. Bringing this filtered data back to the time domain using an inverse S-transform then yields wave-type specific seismograms for all six components (translation and rotation), as shown in Figure 6 for the vertical translational component (where not stated otherwise). The East component of translational motion is displayed for the separated SH-/Love wave data, since SH-/Love energy should not be present on the vertical translation component. Colored lines indicate the onset times of different key phases as obtained from ray tracing in the reference PREM model. Note that several key phases appear to exclusively show up in the expected separated components. Such decomposed seismograms can significantly facilitate the interpretation of single-station seismic recordings and enable the application of wave-type specific imaging schemes. In the future, such wave-type classification and separation schemes could be used to extract certain waves from ambient noise recordings, given that a sufficiently sensitive rotational sensor exists.

### 3.2. Sparse Wavefield Sampling

*“I do not see that land equipment and exploration can expect a bright future if oil continues to hover around its current price. However, this is only if land seismic carries on in the way it has been doing. If we make some overdue technological changes and adhere better to the science, the future is bright and potentially very profitable.”*

**Bob Heath (2018)** [71].

**Figure 7:**
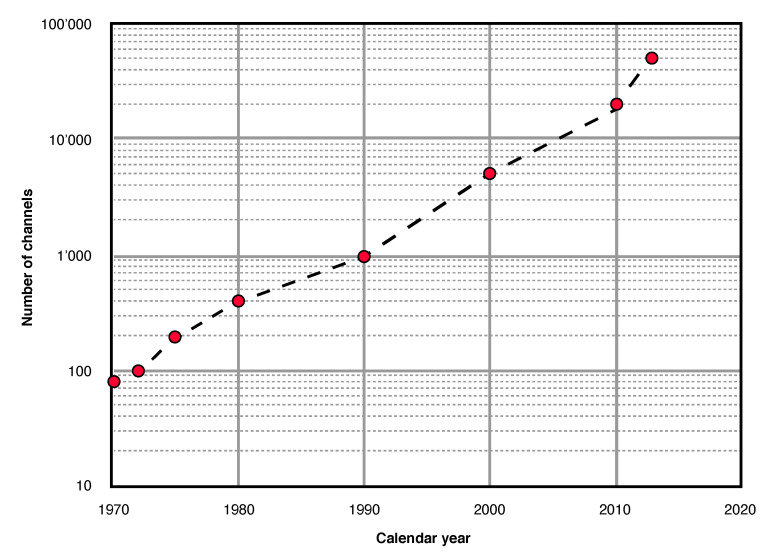
History of land seismic recording system channel count versus calendar year modified after Reference [72].

In a recent commentary, Bob Heath paints a grim picture for the future of the land seismic exploration industry [71]. He argues that over the last years, manufacturers and contractors in the industry adhered to a ‘brute-force’ mentality and, instead of listening to science and innovation, simply tried to overcome acquisition challenges by increasing the seismic channel counts. And indeed, the number of recording channels used in the land seismic exploration industry has been steadily increasing over the last decades, as shown in Figure 7 [72]. Land exploration activities are thus extremely costly operations involving vast amounts of equipment and personnel. In times of low oil prices, these operations are not profitable anymore. The main benefit of 6DOF measurements for land seismic exploration can thus be identified in that they allow for sparser acquisition compared to conventional acquisition with geophones, thus potentially reducing the costs of land seismic operations. In this section, we will discuss how rotational data can be used to relax spatial sampling requirements, thus leading to a reduction of the number of stations required.

The rules dictating the spatial sampling of a seismic wavefield are governed by the Nyquist–Shannon sampling theorem [73], stating that, in order to reconstruct a wavefield from a set of discrete, regular spatial measurements at spacing Δx, the wavefield must contain no information at and above the Nyquist wavenumber $k_{ny}=1/\left(2\Delta x\right)$. Low-velocity surface waves are characterized by high wavenumbers and thus often require the spatial sampling interval Δx to be small. However, the Nyquist–Shannon sampling theorem assumes sampling of a single quantity of the wavefield. If multiple data types, corresponding to data filtered before sampling with linearly independent filters in the domain of sampling are available, then the Nyquist–Shannon sampling criterion is relaxed proportionally to the new degrees of freedom added to solve the problem. This so-called generalized sampling theorem formulated by Papoulis [74] provides the mathematical framework to describe the power of rotational measurements for wavefield reconstruction.

**Figure 8:**
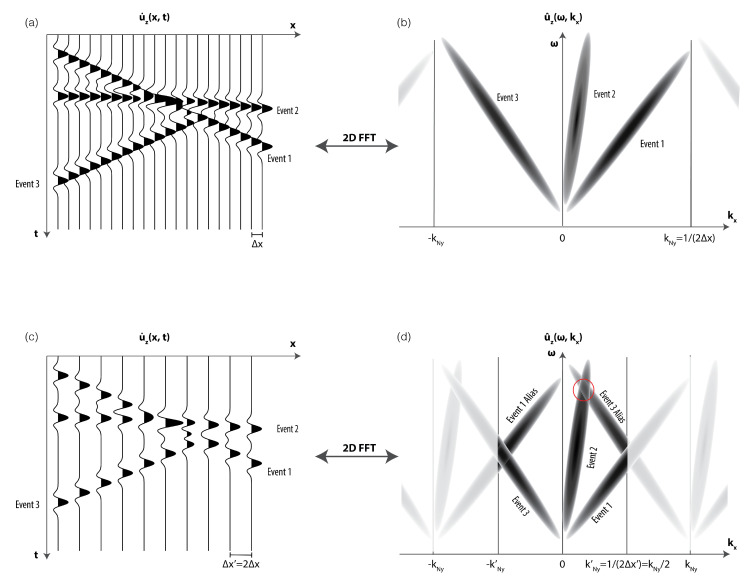
Spatial aliasing in the frequency-wavenumber domain (ω-*k*-domain). The top two panels (**a**,**b**) show a wavefield that is sampled according to the Shannon-Nyquist criterion. The wavefield in the bottom two panels (**c**,**d**) is sampled at half the Nyquist-wavenumber leading to aliasing. The red circle marks a region where aliased and unaliased data overlap in the ω-*k*-domain.

Let us consider a simple seismic survey, such as the one depicted in the example in Figure 8. The vertical-component seismic wavefield ${\dot{u}}_{z}\left(x,t\right)$ is recorded with a linear array of vertical-component geophones spaced at an interval of Δx (Figure 8a). In our example, the wavefield contains three distinct events: event 1 showing a linear moveout as it would be observed for a surface wave, event 2 showing a hyperbolic moveout as it would be expected for a reflected body wave, and event 3 showing the same linear moveout velocity as event 1, but propagating in the opposite direction (e.g., as expected for a back-scattered surface wave). Applying a two-dimensional Fourier transform to the data will yield the recorded wavefield ${\hat{u}}_{z}\left(\omega ,k_{x}\right)$ in the frequency-wavenumber- ($\omega ,k_{x}$-) domain (Figure 8b).

If the geophone spacing Δx is small enough (top two panels in Figure 8), all signal in the ω–$k_{x}$-domain will sit within a cone-shaped region in between the positive and negative Nyquist-wavenumber, where the width of the cone is determined by the events with the lowest moveout velocity in the recorded data (events 1 and 3 in the given example). In this case, no information is contained at or above the Nyquist-wavenumber $k_{Ny}$, and the wavefield in the space-time domain $\dot{u}\left(x,t\right)$ can be reconstructed at an arbitrary small spacing using the Shannon-Nyquist sampling theorem. Due to discrete sampling, the signal cone is periodically repeated infinitely many times along the $k_{x}$ axis at a spacing of $2k_{Ny}$. These so-called aliases are hinted at in the faded part of the top right panel of Figure 8. The reconstruction is now achieved, by multiplying the data in the ω–$k_{x}$-domain with the following box function:

(27) $$\Pi \left(\omega ,k_{x}\right)=\left\{\begin{array}{c} 0,\mathrm{if}\;\left|k_{x}\right|>k_{Ny}\\ \frac{1}{2},\mathrm{if}\;\left|k_{x}\right|=k_{Ny}\\ 1,\mathrm{if}\;\left|k_{x}\right|<k_{Ny} \end{array}\right.,$$

thereby suppressing the aliases. This filtering process corresponds to a spatial convolution with a sinc function in the *x*-*t*-domain [73] resulting in a perfect reconstruction of the wavefield at an arbitrary small sampling interval.

In the bottom two panels (Figure 8c,d), the geophone spacing is now increased by a factor of 2 with respect to the example in the top two panels (Figure 8a,b), leading to a Nyquist wavenumber $k_{Ny}^{\prime }$ that is only half as big. The recorded data is now spatially aliased, meaning that the periodically repeated signal cones along the wavenumber axis overlap for the low velocity events (events 1 and 3) as depicted in Figure 8d. As a result, aliased and unaliased data are super-imposed in the frequency-wavenumber-domain (e.g., in the area marked with a red circle) and no reconstruction on a finer sampling interval can be achieved using the sinc filter described above.

Now, assume that at each position where we have a vertical component geophone, we additionally record the wavefield with a rotational sensor, measuring the rotational motions ${\dot{\omega }}_{y}$ around the crossline (y-) axis (horizontal axis orthogonal to the x-axis). Due to the free-surface boundary condition, the rotational sensor will directly measure the inline horizontal derivative of the vertical translational wavefield component: ${\dot{\omega }}_{y}=-\partial_{x}{\dot{u}}_{z}$ (Equation (19)). The rotational sensor thus allows us to directly measure the wavefield gradient. Such a gradient measurement is a spatially filtered version of the vertical component data that can be used to apply the generalized sampling theorem after Papoulis [74] in order to reconstruct the vertical component wavefield. In the frequency-wavenumber domain, this spatial derivative filter is simply described by a multiplication with the factor $-jk_{x}$, with *j* being the imaginary unit:

(28) $$\partial_{x}{\dot{u}}_{z}\left(x,t\right)⟷-jk_{x}{\hat{u}}_{z}\left(\omega ,k_{x}\right).$$

In the aliased regions of the ω-$k_{x}$-spectrum, the data will be the sum of the unaliased part ${\hat{u}}_{z}\left(\omega ,k_{x}\right)$ and aliased part ${\hat{u}}_{z}^{\prime }\left(\omega ,k_{x}\right)$ (see the red circle in Figure 8). For the two-component data recorded by the vertical component geophone ${\dot{u}}_{Z}$ and the crossline rotational sensor ${\dot{\omega }}_{y}$, we can thus set up the following set of linear equations [75]:

(29) u˙z(ω,kx)ω˙y(ω,kx)=11−jkx−j(kx−2kNy′)u^z(ω,kx)u^z′(ω,kx),

where $k_{Ny}^{\prime }$ corresponds to the Nyquist wavenumber of the aliased survey shown Figure 8c,d. Equation (29) can now be solved to yield the unaliased and aliased parts ${\hat{u}}_{z}\left(\omega ,k_{x}\right)$ and ${\hat{u}}_{z}^{\prime }\left(\omega ,k_{x}\right)$, respectively. This effectively enables us to double the Nyquist-wavenumber by simply extending the wavenumber range with the recovered aliased part at the reduced Nyquist-wavenumber. As a result, we can now fully reconstruct the wavefield shown in the bottom two panels in Figure 8 using only half as many spatial sampling positions as conventionally required when only vertical component geophone data is available. Note that generalized sampling does not result in a reduction of the number of acquisition channels (the total amount of acquired data remains the same), but rather in a reduction of the number of spatial sampling points.

If both the rotational sensor and the geophone are installed within the same sensor housing, the number of sensors required to properly sample the wavefield can thus effectively be halved. This is of particular importance for 3D seismic exploration campaigns, where the wavefield is additionally sampled in the crossline direction, leading to installations involving vast amounts of receivers. Since the deployment of sensors in the field (cost of installation) is one of the major factors driving the costs and length of a seismic exploration campaign, rotational sensors can help to significantly reduce the required efforts. Similar techniques are already successfully applied in marine seismic streamer acquisition incorporating combined recordings of the pressure and particle velocity (which is proportional to the spatial gradient of the pressure data) in all three spatial directions [76,77].

**Figure 9:**
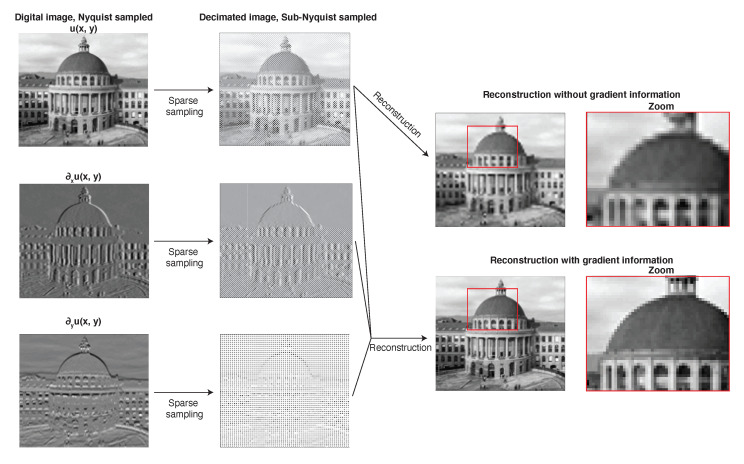
Image reconstruction of a picture of the ETH main building illustrating the power of using gradient information in reconstruction problems. The same idea can be applied to reconstruct seismic wavefields, if measurements of the wavefield’s gradients (e.g., due to a rotational sensor at the Earth’s surface) are available.

In order to illustrate the power of having access to gradient information when solving reconstruction problems, we apply the reconstruction scheme described above to an image of the main building of ETH Zürich. The results are shown in Figure 9. The original image (sampled at the Nyquist rate) and its gradient (derivatives in x- and y-directions) are shown in the leftmost panels. Now, assume that we only have access to every second pixel of both the image and its gradient (middle panels). If we try to interpolate the image back to its original resolution without using the gradient data, we only obtain a blurred reconstruction showing aliasing (top two panels on the right). If we now incorporate the gradient information and reconstruct the image using Equation (29) (assuming the image pixels are our vertical-component geophone data and the pixels of the two gradients of the image our horizontal rotational data), we obtain the image in its original resolution (bottom two panels on the right). Naturally, the differences are most noticeable in areas where the picture shows strong spatial variations (i.e., its gradient is different from zero). This is most pronounced in the windows and pillars of the building and at edges.

As discussed in Section 3.1.2, the dominating feature in land seismic data dictating the spatial sampling is coherent surface wave noise, which exhibits the shortest wavelengths of all typically recorded wave types. If single-station methods, such as the wave mode filtering algorithm described in this paper, can be applied to suppress this noise, the spatial sampling can potentially be significantly relaxed. Currently, such algorithms are computationally demanding and can therefore not be applied to large data volumes, such as typically encountered in seismic exploration. Thus, there is a need for the development of efficient algorithms that enable 6DOF wave mode filtering for large datasets. We foresee that the recent, rapid emergence of machine learning methods will play an important role in enabling such algorithms. Even if such algorithms can be realized, conventional array processing methods will likely have the advantage of providing a better signal-to-noise ratio when compared to single-station approaches due to the possibility to stack signals from individual array elements. We, therefore, expect that an optimal filtering approach will consider a combination of single-station and array processing methods for wave mode filtering.

At present, there are some economical and logistical limitations that exacerbate the use of rotational sensors in seismic exploration. Most of the sensors that are available to date are specifically designed for the needs in global seismology (see Section 4). The high demands on sensitivity dictated by global seismology led to devices that are bulky and heavy (for example, due to the need for very long fibre loops to reach the required sensitivity for fibre-optic gyroscopes). Additionally, most of the available systems include analog-to-digital converters and electronics for remote single-station deployment, leading to a high power consumption and high costs per unit. This renders most of the devices unsuitable for application in land seismic exploration. For wide-spread applications of rotational motion sensors in seismic exploration, it is therefore crucial to bring down the costs per unit. Since signal levels are expected to be much higher in seismic exploration than in global seismology, due to the proximity to the source [21], smaller and less sensitive devices with an analog output should be envisaged. Innovative rotation sensors based on the principles of conventional geophones, such as described by Muyzert et al. [78], could prove a valuable alternative to expensive fibre-optic gyroscope for wide-spread use in land-seismic exploration.

### 3.3. Rotational Data as a New Observable to Constrain Inverse Problems

Geophysicists are often faced with optimization problems that are severely under-determined, meaning that the number of data points that are available is much smaller than the number of parameters that need to be estimated. An example is the seismic tomography problem, which can often only be solved by enforcing strong regularization (i.e., a priori knowledge). Any additional data that can be obtained, such as rotational data, should thus help to better constrain the problem. Indeed, it was shown that the additional data obtained by rotational motion measurements can help to reduce some of the ambiguity of seismic inverse problems. In this section, we review recent progress in the use of rotational data to constrain seismic inverse problems.

Since rotational data are gradient measurements, they are mainly sensitive to small-scale heterogeneity (i.e., large values are measured where the wavefield changes rapidly in space) [79]. This local sensitivity can help to better constrain the near-receiver structure. Fichtner and Igel [80] derived sensitivity densities for combined measurements of translational and rotational ground-motion and demonstrated that such kernels only attain large absolute values in the vicinity of the receiver, independent of the source position, magnitude and timing. Based on this work, Bernauer et al. [81] and Bernauer et al. [82] proposed to incorporate rotational motion recordings into seismic tomography to yield better resolved and more realistic tomographic images of the near-receiver structure without the requirement to know the deeper structure.

The use of rotational motion measurements was also demonstrated to reduce the non-uniqueness in finite source inverse problems [83,84] and to improve the characterization of the seismic moment tensor [85]. The incorporation of rotational motions into the source inversion schemes could stabilize results with only half the amount of stations required to achieve comparable results using conventional pure translational data.

Rotational motions are also considered in long-period seismology. Igel et al. [86] reported on the first direct observations of the toroidal eigen-modes of the Earth using the Wettzell ring laser. Further analysis of these ring laser observations of the Earth’s free oscillations also enabled the detection of energy from spheroidal oscillations that could potentially help to better constrain the deep interior properties of the Earth [87,88].

Limited studies exist on the potential of using rotational data for imaging. Li and van der Baan [89] demonstrated that the incorporation of rotational motion helps to enhance imaging of microseismic source locations.

Even though rotational motion seismology is still in its infancy, all of the studies above indicate that a significant information gain can be achieved when incorporating rotational motions into geophysical inverse problems. Most of the studies listed above are purely theoretical and a demonstration of the benefits of rotational motion measurements with field data are missing. Given that rotational sensors are now becoming more wide-spread, we expect that this gap will be closed in the near-future and new schemes will be developed that will incorporate rotational motions into, for example, full waveform inversion or seismic interferometry (see theoretical foundations in [90]).

### 3.4. Tilt Corrections of Translational Data

Conventional inertial seismometers are subject to rotational motions induced by the passing of seismic waves. This leads to changes in the projection of gravity onto the three inertial components. Direct measurements of the effective rotation permit correcting the recorded seismograms for these effects. Tilt effects are especially significant for long period signals. Various authors discuss the deteriorating effects that rotations (particularly tilt) can have on the recordings of conventional inertial seismometers [91,92,93,94] and suggest potential correction schemes [9,93,95,96,97]. Tilt contamination of inertial seismometer recordings has been shown to be significant, for example, in the near field of active volcanoes [98] and on the ocean bottom [28] and was demonstrated to have an adverse effect on moment tensor inversions [98].

### 3.5. Earthquake Engineering

For centuries, earthquake seismologists have been arguing about the importance of rotational effects associated with earthquakes. As summarized by Ferrari [99], there are various reports on the observations of twisting of tombstones, statues and monuments after earthquakes, e.g., Reference [100,101]. Even though most of these effects can be explained to result from pure translational ground-motion and are likely caused by the asymmetry of the constructions, there are indications that this is not always the case [15,102]. In fact, the structural response of buildings to rotational motions is widely unknown [103]. Despite indications that damage caused by rotational motions might be significant [104,105,106,107,108,109,110], earthquake hazard assessment was until recently almost exclusively based on the analysis of translational motion. Especially in the near-field of earthquakes, rotational motion amplitudes can be significant, e.g., Reference [5,111,112], with a large potential for damage. Hence, there is a large interest by the earthquake engineering community to develop reliable rotational sensors for the evaluation of rotational motions induced in buildings and for the estimation of infrastructural damage risk caused by rotational ground-motions.

Zembaty et al. [113] showed that rotational motion data enables the estimation of the stiffness of a material in structural health monitoring.

## 4. How to Measure Rotational Motions

Even though the main focus of this paper is the processing of rotational seismic data, in this section, we will briefly review the concepts of how rotational ground-motion can be measured. This is mainly to give the interested reader an overview over the technology that is currently available. Measurements of rotational ground motion can either be obtained from (1) direct observations using a dedicated rotational sensor or (2) estimated from dense arrays of conventional inertial sensors using a finite-difference approximation.

### 4.1. Direct Observations

The development of dedicated rotational sensors for seismological applications is challenging since specific requirements need to be fulfilled. (1) Rotation sensors require a high sensitivity over a broad frequency-range, since ground rotation rates are expected to be in the range of $10^{-14}$ rad/s to 1 rad/s [10,114]. (2) A rotational seismometer must be insusceptible to linear translational motions. (3) Self-noise levels need to be temperature-independent. (4) The sensor must be stable against magnetic field variations. A variety of technical solutions have been proposed, which we will briefly review in the following.

The most sensitive instruments for ground rotation sensing are large ring laser gyroscopes. Originally developed to monitor the Earth’s absolute rotation rate, these sensors also provide data of rotational ground-motions generated by earthquakes and ocean noise, e.g., Reference [9,34]. Ring lasers are so-called Sagnac interferometers that detect the beat frequency of two counter-propagating laser beams [9,115,116]. This beat frequency is directly proportional to the rotation rate. Just recently, in 2017, the first-of-its-kind multicomponent ring laser gyroscope ROMY started delivering first observations of rotational ground-motions [66].

While ring lasers provide highly accurate measurements of ground rotational motion, the sheer size and cost of such devices excludes them from widespread use in seismology and seismic exploration. Thus, there is a need for portable instruments. The most common technologies for portable rotation sensors are liquid-based systems, magneto-hydrodynamic sensors, and fibre-optic gyroscopes.

Liquid-based rotational motion sensors sense the differential movement in a liquid-filled torus by means of molecular electronic transducers (MET) [117,118]. Such sensors were successfully employed to record rotational ground-motions generated by nearby artificial sources [16,119,120] but show high sensitivity to changes in ambient temperature [121].

Magneto-hydrodynamic sensors make use of the inertia of a conductive fluid in a stable external magnetic field to measure angular motions, e.g., Reference [122]. The relative motion between the fluid and the magnetic field in case of externally applied rotation generates a radial current that can be measured.

Fibre-optic gyroscopes are based on the same principle as large ring lasers. The Sagnac effect (beat frequency of two propagating laser beams) is evaluated inside a fibre-optical loop [116]. A major advantage of fibre-optical sensors is that they are inherently insensitive to translational motion. Fibre-optic gyroscopes were already successfully employed for seismological applications [28,123,124,125,126,127]. Recently, the first commercial fiber optic gyroscope designed specifically for the needs of seismology has become available blueSeis—3A by iXBlue, France [12].

D’Alessandro and D’Anna [128] and Liu and Pike [129] propose rotational sensors based on micro electromechanical system (MEMS) technology. Brokešová and Málek [13] mounted conventional geophones on a fixed frame and used a finite-difference approximation to estimate spatial derivatives of the particle velocity field that can be used to compute rotation rates around all three coordinate axes. Their sensor, called rotaphone, has since successfully recorded rotational ground-motions in tectonically active regions around the globe [130]. Muyzert et al. [78] proposed a rotational sensor consisting of a pair of 3C microelectromechanical accelerometers that are vertically placed a few centimeters apart within a cylindrical housing. If this sensor is planted vertically just below the Earth’s surface, the vertical gradient between the two sensors is enough to measure the two horizontal components of rotational motions due to the free surface boundary condition (Equation (19)). This relatively cheap implementation makes the sensor suitable for mass production and use in seismic exploration.

Barak et al. [131] recognized that conventional induction-coil magnetometers can potentially be used to measure ground rotational motions since a rotation of the sensor results in a change in the projection of the Earth’s magnetic field on the three orthogonal magnetometer components.

Under certain assumptions, rotational motions can also be estimated from a single recording of translational motion by decomposing the three-component seismograms into body waves [132].

### 4.2. Array-Derived Rotational Motions

Since reliable rotational instruments have not been available until recently, arrays of conventional three-component inertial sensors have been widely used to estimate rotational motion by computing the horizontal spatial gradients of the recorded seismic wavefield measured at the Earth’s surface, e.g., Reference [29,43,44,45,133,134,135,136]. Techniques to estimate spatial wavefield gradients from arrays of 3C stations are generally based on the expansion of the wavefield about a central (master) station using a Taylor series. This allows one to estimate the complete displacement gradient tensor (Equation (2)) and thus also permits the estimation of ground strains. Suryanto et al. [136] compared array-derived rotational motions with direct ring-laser observations and found a surprisingly good fit (correlation coefficient of 0.94). However, the Taylor series approximation is only reasonable under the assumption that the subsurface is homogeneous and deformation varies linearly over the array area. In addition, array-based spatial wavefield gradient data is prone to noise caused by measurement uncertainties, such as varying receiver-to-ground coupling, differences in the instrument response, or sensor orientation [137,138,139,140,141]. Further issues with array-derived rotation estimates can be caused by strain-rotation coupling, a site-effect describing the conversion of strains on a large scale (seismic wavelength) to rotations on a local scale due to near-receiver heterogeneity [142].

## 5. Conclusions

We anticipate that dedicated rotational sensors will soon become both more accurate and more affordable. This could eventually change the standard instrumentation procedures in seismology from conventional inertial seismometers to full 6DOF setups. In order to help seismologists to embrace rotational motions as new observables, it is necessary to extensively illustrate the benefits of such measurements with field data examples. In this paper, we provide the first-ever 6DOF analysis of a teleseismic earthquake recorded on a multicomponent ring laser, illustrating the rich amount of information on wave parameters and wave type that can be extracted from only a very limited dataset (a single source and a single recording station).

We, therefore, expect that 6DOF seismometers will play an important role for seismology in areas where the employment of large source-receiver configurations is not possible. This is especially true in extraterrestrial seismology where logistical constraints limit the amount of sensors that can be employed and seismic events are rare due to the absence of plate tectonics on most planets and moons in our solar system. Here, 6DOF ground-motion data can help to extract the most information from the limited amount of available data and ultimately lead to a better understanding on the interior structure and formation of extraterrestrial objects. The development of 6DOF ground-motion sensors for space exploration is currently underway [143].

In land seismic exploration, rotational data allow one to relax the spatial sampling requirements inherent to conventional seismic acquisition, allowing for sparser receiver layouts and resulting in a significant reduction of the number of receivers needed in land seismic operations.
